# The Therapeutic Evaluation of Spinal Canal Decompression by Using the TBEIS Technique in the Treatment of Lumbar Spinal Stenosis

**DOI:** 10.1155/2020/6183027

**Published:** 2020-05-21

**Authors:** Kemin You, Bo Li, Hongze Chang, Yan Zhang, Feng Cai, Liang Liu, Xiaodong Liu

**Affiliations:** Department of Orthopedic Surgery, Yangpu Hospital, Tongji University School of Medicine, 450 Tengyue Road, Shanghai 200090, China

## Abstract

**Objective:**

To evaluate the clinical efficacy of the percutaneous endoscopic Transforaminal Broad Easy Immediate Surgery (TBEIS) technology in elderly patients with lumbar spinal stenosis (LSS).

**Methods:**

From February 2016 to May 2018, 35 elderly patients with LSS were treated with the TBEIS technique. There were 23 males and 12 females, aged from 53 to 72 years with a median age of 63.1 years. Preoperative, 1 day, and 1 and 12 months postoperative visual analogue scale (VAS) scores and Oswestry Disability Index (ODI) were statistically analyzed. The modified MacNab criterion was used to assess the clinical effects. The radiological outcomes were evaluated by X-ray and computed tomography (CT).

**Results:**

All of the operations were successful. The operative time ranged from 120 to 170 min with a median time of 148 min. All of the patients were followed up for 12 to 38 months with a median follow-up of 18 months. Preoperative, 1 day, and 1 and 12 months postoperative VAS leg scores were 6.91 ± 0.98, 1.69 ± 0.68, 1.23 ± 0.59, and 0.91 ± 0.61, respectively, and the VAS back scores improved from 4.51 ± 0.82 to 0.66 ± 0.68. The ODI scores were 63.82 ± 7.59, 38.79 ± 6.36, 24.79 ± 3.90, and 11.33 ± 3.92, respectively. Postoperative scores of VAS and ODI were obviously improved (*P* < 0.01). According to the modified MacNab criteria used to evaluate the clinical effects, 11 cases achieved excellent results, 18 cases achieved good results, 4 cases achieved fair results, and 2 cases achieved poor results. There were no neurovascular injury and other complications.

**Conclusions:**

Treatment of LSS in the elderly patients by the TBEIS technology has good clinical efficacy, and the technique is safe and minimally invasive.

## 1. Introduction

Lumbar spinal stenosis (LSS) is a common spinal disease in the elderly. Some of them usually have an infirm condition, and traditional open surgery is not applicable to this group of patients [1, 2]. In recent years, the development of minimally invasive spinal technology has overcome the disadvantages of traditional surgery and can better preserve the integrity of the posterior ligament complex of the spine [3, 4]. Yeung [5, 6] created the Yeung endoscopic spine system (YESS) on this basis. Hoogland et al. [7] proposed the transforaminal endoscopic spine system (TESSYS) based on YESS. Currently, TESSYS technology and improved TESSYS technology have become increasingly popular in treating different types of lumbar disc herniation (LDH) [8–12].

LSS is usually accompanied by degeneration of intervertebral disc and facet joints, the relaxation of ligamentum flavum and posterior longitudinal ligaments. Eventually, the superior articular process (SAP) dislocates forward and upward, and the dural sac and the nerve roots are subjected to circumferential compression.

To solve the above problems, our research team applied YESS, TESSYS, and improved TESSYS technologies, such as target point technology, Under Laminar Lumbar Endoscopic Spinal System (ULESS) [13], and Transforaminal Broad Easy Immediate Surgery (TBEIS), to treat patients of different ages who had lumbar intervertebral disc herniation at different herniation sites and achieved good results. On this basis, our research team tried to perform the treatment of patients with LSS by TBEIS that decompresses the LSS from the dorsal side to the ventral side or from the ventral side to the dorsal side according to the location and degree of the LSS and observed the effect of this operation and analyzed its feasibility.

## 2. Materials and Methods

### 2.1. Ethics Statement

This study was approved by the Ethics Committee of Yangpu Hospital affiliated to Tongji University and was performed in accordance with the Helsinki Declaration. Written informed consent was not required for this type of study. All of the data were analyzed anonymously.

### 2.2. Patients

This retrospective study reviewed 35 consecutive patients with LSS. The inclusion criteria were as follows: (1) patients who were diagnosed with degenerative LSS (central spinal canal stenosis, lateral spinal stenosis, foraminal spinal stenosis, or any combination of these conditions) with moderate to severe stenosis shown on cross-sectional MRI or CT scan images [14]; (2) patients who were diagnosed with/without disk herniation; (3) patients who complained of intermittent neurogenic claudication with/without radicular leg pain; and (4) patients who accepted conservative treatments (medications or physical therapy) for more than 6 months. Most scholars believe that surgical treatment showed greater benefits in patients with moderate to severe LSS compared with conservative treatment [15], and therefore, only patients with grades moderate and severe were included in our study. The severity of LSS based on morphology of the dural sac on axial magnetic resonance imaging (MRI) was used in our study. The severity of LSS was divided into 4 grades (no stenosis, mild stenosis, moderate stenosis, and severe stenosis) [14]. Moderate stenosis is characterized by moderate obliteration of the anterior Cerebrospinal Fluid (CSF) space and aggregation of some nerves in the cauda equina, making it impossible to visually separate them. Severe stenosis is defined as severe obliteration of the anterior CSF space causing marked compression of the dural sac and inability to visually separate the nerves in the cauda equina from each other, thus appearing instead as one bundle. All of the operations were carried out by the same spine surgeon at the Yangpu Hospital. The contraindications were as follows: (1) patients with segment instability (more than grade II spondylolisthesis [16, 17] or dynamic instability [17]); (2) patients having inoperable medical disease; (3) stenosis of more than two segments; and (4) patients with peripheral nerve disease.

### 2.3. Operative Technique

All of the procedures were carried out using the transforaminal endoscopic spine system (Joinmax, Karlsruhe, Germany).

**Figure 1:**
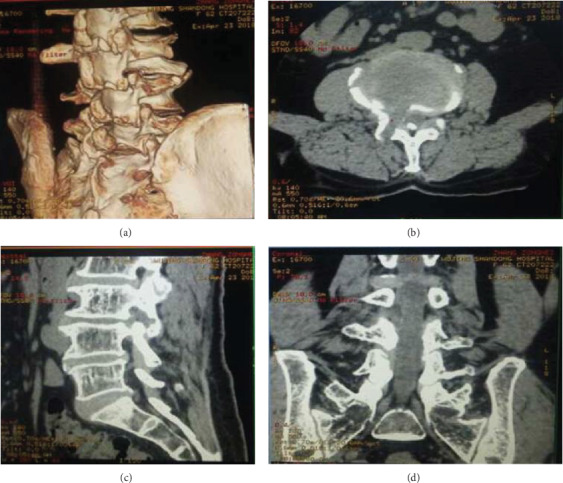
An illustrative case of foraminoplasty assessed by a 3D spiral CT scan during the operation at the L3-4 level.

**Figure 2:**
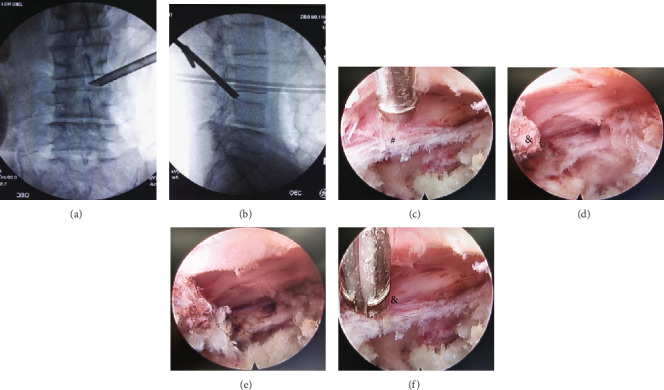
Radiographs showing that the working cannula has been successfully inserted into the surgical area at the L3-4 level: (a) anteroposterior radiographs and (b) lateral radiographs. Endoscopic view of the surgical procedure: endoscopic views of (c) before and (d) after removal of the ligamentum flavum and the extruded disc. Endoscopic views of (e) before and (f) after removal of the hyperplastic facet joint to relieve compression from the lateral recess.

**Figure 3:**
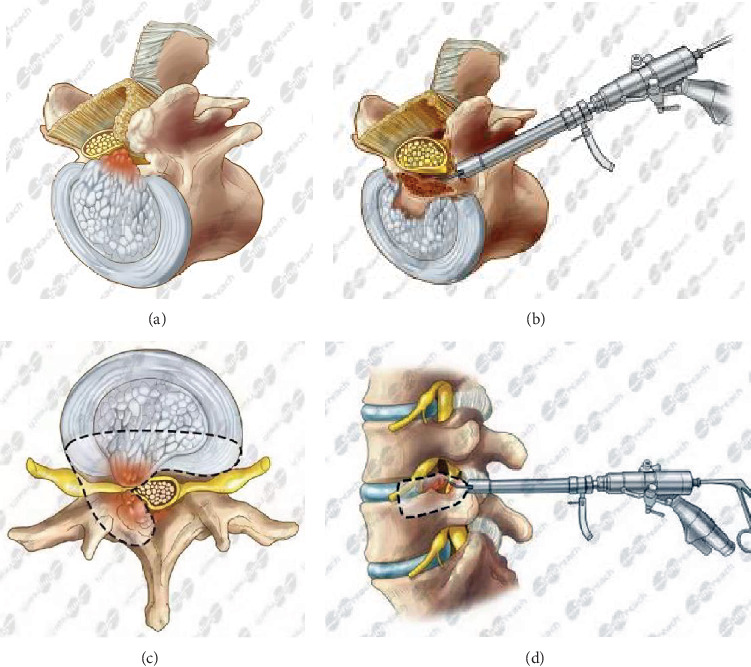
Schematic diagram of the decompression range of the spinal canal. In the axial view, the decompression range includes the front space of the entire spinal canal and the rear space of the ipsilateral spinal canal (a–c). In the sagittal view, the decompression range of the canal height starts from the exiting root of the same segment to the upper third of the lower vertebra (d).

1. Preoperative localization: the patient was asked to lie prone on the operating table. The puncture point was about 8-10 cm away from the spinous process line at L3/4, 12-14 cm at L4/5 and L5/S1. A Kirschner wire was used as a reference to determine the puncture point under X-ray fluoroscopy. In the lateral view, the Kirschner wire was on the line between the base of the SAP and the posterior superior margin of the vertebral body at the lower level of the lesion segment. In the anteroposterior (AP) view, the Kirschner wire was on the line between the base of the SAP and the midpoint of the upper margin of the lower vertebra, and the intersection point of the two lines was the puncture point
2. Expansive plasty of the foramen and lateral recess (see Figure 1): 0.75% lidocaine was used for local anesthesia after disinfection; in the patients who were selected to undergo decompression of the ventral side of the spinal canal firstly, an 18-gauge access needle was inserted into the SAP at an angle of 25°-30° to the horizontal plane under fluoroscopy guidance, the abduction angle was 25°-35°, and the needle was tilted 25°-35° toward the head. The puncture trajectory was performed according to the direction of the line drawn during the determination of the puncture point previously. The tip of the needle was closely in contact with the anterior and lateral aspects of the SAP. Local anesthesia was performed again after the needle was positioned. In the patients who were selected to undergo decompression of the dorsal side of the spinal canal firstly, an18-gauge access needle was punctured into the space between the dorsal side of the ligamentum flavum and the lamina at the lesion segment. The ligamentum flavum was not broken during the puncture to avoid the damage to the dural sac. A skin incision of about 0.8 cm was made at the puncture point, the tapered guide rod was inserted along the guide wire, and the guide tube was inserted to expand and loosen the soft tissue. The guide rod and guide tube were placed and adjusted continuously according to the type and extent of LSS. Different models of reamers were inserted to grind the base of the SAP and trim the bone surface. For patients with severe LSS, the working channel established by manual reamer in foraminoplasty may no longer meet the requirements. At this time, the bone of facet joints can be directly ground away circularly under the eccentric endoscopic drill, which widens the working channel and enlarges the lateral recess. Part of the isthmus and the inferior articular process (IAP) were removed if necessary. The inclination angle to the head was increased when the foramen was enlarged, and the puncture trajectory was moderately adjusted according to the base line between the SAP and the posterior margin of the lower vertebra in the lateral view. The guide rod reached the posterior border of the vertebral body in the lateral view, and the guide rod reached the spinous process line in the AP view (see Figures 2(a) and 2(b))
3. Installation of the working cannula: hypertrophy of the ligamentum flavum and cohesion of the articular processes significantly led to the application of the decompression sequence similar to that in open surgery from the dorsal to ventral side. If disc herniation was large or the internal volume of the spinal canal was still acceptable, the decompression sequence from the ventral side to the dorsal side was applied. After the working cannula was in proper position, the endoscopic system was introduced into the spinal canal through the working cannula
4. In patients who underwent decompression from the dorsal side firstly, the first endoscopic view field was the ligamentum flavum. The endoscope was retracted, and the cannula was rotated 180° into the ventral aspect of the dural sac and nerve root after decompression of the dorsal side. The endoscope entered into the retrodisc space, and the disc tissue was routinely removed in patients with disc herniation. In patients who underwent ventral decompression firstly, the first endoscopic view field was a “sandwich” structure consisting of the ligamentum flavum, the contents of the spinal canal, and the intervertebral disc. The protruding intervertebral disc was removed first, so that the nerve root fell back and the gap between the ligamentum flavum and the nerve root was enlarged; osteophytes, calcified posterior longitudinal ligament, and intervertebral disc were removed by endoscopic drill. Thus, the dorsal side, the ventral side, and the lateral side of the dural sac and nerve root were decompressed at 270° (see Figure 3)
5. The direction of the working cannula was adjusted to explore the spinal canal to avoid tissue residue; bipolar radiofrequency was used for ablation and hemostasis. At the end of the procedure, the dural sac and nerve root showed obvious spontaneous pulsation, and the blood vessels on the nerve surface were filled (see Figures 2(c)–2(f))

### 2.4. Postoperative Management

After surgery, the patients were instructed to rest in bed on a firm mattress for at least 8 hours. Bending over and lifting weights was prohibited for 6 weeks after surgery. The lumbar brace was removed after 6weeks.

### 2.5. Outcome Measures

**Figure 4:**
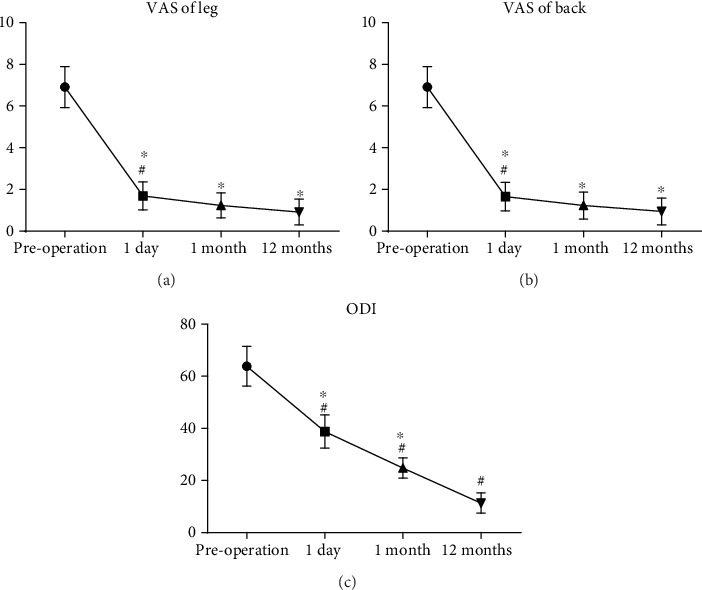
Comparison of (a) VAS scores of the leg, (b) VAS scores of the back, and (c) ODI at different time points. VAS: visual analogue scale; ODI: Oswestry Disability Index. ∗*P* < 0.01 versus the preoperation group. ^#^*P* < 0.01 versus the corresponding last follow-up group.

The radiological outcomes were evaluated by X-ray and CT (see Figure 4). The clinical outcomes were assessed by median VAS [18, 19] and ODI [19] and modified MacNab criteria [20]. Walking distance and standing time were also measured.

### 2.6. Statistical Analysis

The Student's *t* test and Mann–Whitney *U* test were performed to analyze the variables by SPSS 17.0 software (SPSS Inc., Chicago, USA). *P* value < 0.05 was considered to be significant.

## 3. Results

### 3.1. Preoperative Demographic Characteristics and Outcomes

The median duration of symptoms was 19 months. A total of 23 males and 12 females were enrolled. There were 6 cases with grade I spondylolisthesis and no instability on the dynamic X-ray. There were 23 cases with stenosis at L4/5 and 12 cases with stenosis at L5/S1. There were 15 cases with central spinal canal stenosis, 12 cases with lateral spinal stenosis, and 8 cases with stenosis of the central spinal canal and lateral spinal canal. There were 24 cases with grade moderate and 11 cases with grade severe. The median operation time was 140 min, and the blood loss was 25.3 ml. The median hospital stay was 6 days (see Table 1).

### 3.2. Clinical Results

Modified MacNab criteria were applied, and the good-to-excellent rate was 82.8%. Four patients showed fair results, and 2 patients who underwent revision surgery were rated as poor. The details of the outcomes are shown in Table 2.

VAS of the leg decreased from 6.91 ± 0.98 to 1.69 ± 0.68, and it was 0.91 ± 0.61 at the latest follow-up. VAS of the back also decreased from 4.51 ± 0.82 to 1.77 ± 0.60, and it was 0.66 ± 0.68 at the latest follow-up. ODI improved from 63.82 ± 7.59 to 38.79 ± 6.36 immediately after the operation, and it was 11.33 ± 3.92 at the latest follow-up. The details are shown in Table 3 and Figure 4. Both walking distance and standing time increased after the operation (see Tables 4 and 5).

### 3.3. Complications and Recurrence

Complications developed in three patients (8.57%). One patient experienced dysesthesia in the distribution area of the ipsilateral neighboring exiting nerve root. Two cases experienced temporary pain aggravation, and their symptoms were relieved after conservative therapy. One patient developed symptoms of neck pain and chest pain during the operation, and then, the operation was shortly paused; their rogation fluid was stopped and the perfusion pressure was reduced, the body position was adjusted to raise the head position, and the patient was treated with oxygen. Until the patient's symptoms disappeared, we continued the procedure until the operation was completed after the speed of the irrigation fluid was decreased. There were no major complications.

Two patients with sciatica suffered from the same symptom after 6 months. The patient with recurrent sciatica was subjected to transforaminal posterior lumbar interbody fusion (TLIF) when nonsurgical management failed. In this patient, sciatica was relieved until the final follow-up.

### 3.4. Representative Cases

**Figure 5:**
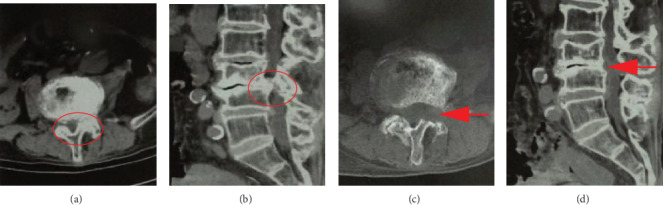
An illustrative case of central spinal canal and lateral recess stenosis causing dorsoventral neural compression. (a) Axial images of preoperative computed tomography (CT) showing central spinal canal and lateral recess stenosis due to ventral compression caused by an osteophytic spur and hyperplasia of the facet joint and extruded disc and dorsal compression caused by hypertrophy of the ligamentum flavum. (b) Sagittal images of preoperative CT examination showing significant stenosis of the spinal canal. (c) Axial images of postoperative CT images showing that the compressive factors were completely removed. (d) Sagittal images of postoperative CT examination showing sufficient decompression of the spinal canal.

Representative case is illustrated in Figure 5.

## 4. Discussion

Elderly patients with LSS often have a long course of illness with severe symptoms and eventually require surgery. In recent years, studies [21, 22] have shown that there is no statistical difference in the therapeutic effect between the endoscopy technology and open spine surgery. The stenotic area in the lateral recess and the intervertebral foramen is just in the surgery area of the percutaneous endoscopic transforaminal approach; hence, the decompression is relatively reliable [23]. For the central LSS of the L5-S1 segment, percutaneous endoscopic surgery through the interlaminar approach can also provide good decompression. However, the remaining lumbar segment is not suitable for an interlaminar approach because of the narrow interlaminar space; hence, it can only be treated through the transforaminal approach. Therefore, our research team has achieved good results with the application of YESS technology, TESSYS technology, and improved TESSYS technology, such as TBEIS technology, to treat patients of different ages with different natures of lumbar disc herniation. On this basis, the application of TBEIS technology in the treatment of LSS in elderly patients was explored. In order to obtain a good clinical effect, decompression was performed from the dorsal side of the spinal canal to the ventral side or from the ventral side of the spinal canal to the dorsal side according to the type and extent of LSS in patients.

As a modified TESSYS technology, this technique emphasizes comprehensive release of the pressure factors on the dorsal and ventral sides of the dural sac and nerve root. TBEIS technology is based on BEIS technology, which was first proposed by professor Bai [24]. This technology is different from YESS and TESSYS technologies. First, during fluoroscopy, the head tilt angle of the puncture needle was increased and the trajectory of the puncture needle was adjusted according to the baseline between the SAP and the posterior margin of the lower vertebral body. Then, the anterior lateral base part of the SAP was removed with a reamer to enlarge the intervertebral foramen. Second, the depth of reamer reaches or exceeds the line of the spinous process in AP view. Third, the core concept of this technique is total decompression of the dorsal and ventral sides of the nerve root and dural sac. Compared with the representative technique of spinal endoscopic technology, such as YESS technology [25] and TESSYS technology [7], the working cannula has a head tilt angle of 60° or even 70°, making the scope of endoscopic exploration and decompression more extensive. A 270° of the whole spinal canal was decompressed, and the effective cross-sectional area and volume of the spinal canal were increased.

Additionally, we changed the place of foraminoplasty from the tip of the SAP to the base of the SAP, which is different from that in the traditional TESSYS technology. YESS or TESSYS technology can also achieve this type of foraminoplasty by changing the puncture site and angle of the head tilt of the reamer. However, the elderly are the majority in LSS patients; the degeneration of facet joints is severe and accompanied by markedly narrow lateral recesses and intervertebral foramen. The TBEIS technique foraminoplasty can avoid damage to the exiting root in the middle of an apparently narrow intervertebral foramen because the location of foraminoplasty is far away from the exiting nerve root. Meanwhile, the introduced protective cannula, which has a duck-mouth-like distal end, could protect the exiting nerve root during the removal of the SAP, and individual foraminoplasty could be performed by manipulating the cannula in different directions. During the operation, the depth of foraminoplasty could be controlled by the stopper, which can be locked at the caudal side of the reamer.

The manual reamer is applied during foraminoplasty, and the main purpose is to establish a working channel that can be performed safely under fluoroscopy. The manual reamer is sufficient if only the nucleus pulposus need to be removed. For patients with severe LSS, it is necessary to adjust the angle and operating range of the working cannula constantly to complete sufficient decompression. The working channel established by manual reamer in foraminoplasty under fluoroscopy can no longer meet the requirements. At this time, the bone hyperplasia of facet joints can be directly ground away under the endoscopic drill, which will widen the working channel and enlarge the lateral recess. In this way, the space of the working cannula is increased, which is convenient for increasing the tilt angle. The use of manual reamer for foraminoplasty is performed under fluoroscopy with high efficiency, while the endoscopic operation is more delicate and safer in the direct vision. The combination of these two methods can make the whole operation safe and effective.

TBEIS technology was an improvement of the TESSYS technology, rather than target point operation [26], and the technique paid more attention to the overall removal of the compression factors in the ventral and dorsal sides of the compressed nerve root and dural sac [27]. There are several standardized requirements for this operation. The endoscopic criteria for the end of operation are as follows. First, the reduction of nerve root and dural sac could be observed after decompression. Second, the blood vessels on the surface of nerve root and dural sac were filled well, and the dural sac, the traversing root, and the exiting root were pulsating obviously. Third, the Lasegue sign was negative and the nerve root slides freely under traction. Many studies [28–32] have found that the long-term efficacy of percutaneous transforaminal endoscopic surgery in the treatment of LSS is stable. The physiological structure of the spine is preserved, there is no need for internal fixation during the surgery, and the cost is less, which greatly enhance the patient's treatment experience compared with the open surgery.

In this study, the follow-up evaluation of patients showed that VAS scores and ODI scores during each postoperative period were significantly lower than those before surgery, which indicates that the TBEIS technology can improve lumbar spine function. We consider that it may be related to the fact that the method does not require removal of the posterior structure of the spine, thereby maximally retaining the structural integrity of the patient's spine and avoiding long-term low back pain and lumbar instability. We have operated 35 patients in more than two years with the TBEIS technique, because patients with LSS are mainly elderly and accompanied by lumbar instability, which is a contraindication to endoscopic decompression alone. Therefore, only 35 patients have been included in our study according to the research criteria so far. 35 patients may be underrepresented; we will continue to expand the number of cases in future studies.

## 5. Conclusions

The study shows that the TBEIS technique can treat elderly patients with LSS and improve the quality of life of patients with infirm bodies. It is an effective minimally invasive surgical method for the treatment of senile degenerative LSS.

## Figures and Tables

**Table 1:** Demographics of this study.

	Median (range) or *n* (%)
Age (years)	63.09 (53-72)
Sex (male)	*n* (65.7)
Duration of symptoms (months)	19.66 (13-28)
Levels involved	
L4-L5	23 (65.7)
L5-S1	12 (34.3)
Type of stenosis	
Central stenosis	15 (42.9)
Lateral recess stenosis	12 (34.3)
Central stenosis combined with lateral recess stenosis	8 (22.8)
Schizas grade C	20 (57.1)
Schizas grade D	15 (42.9)
Number of levels operated	
One level	35 (100)
Two levels	0 (0)
Spondylolisthesis	
Grade 0	29 (82.9)
Grade 1	6 (17.1)
Follow-up (months)	16.00 (12-19)
Blood loss (ml)	25.26 (15-35)
Duration of surgery (min)	140.71 (120-170)
Hospital stay (days)	5.97 (5-7)

**Table 2:** Modified MacNab criteria.

Outcome	Description	*N* (%)
Excellent	Complete relief of symptoms	11 (31.4)
Good	Marked improvement but occasional pain	18 (51.4)
Fair	Improved functional capacity and the need for pain medications	4 (11.4)
Poor	Unimproved symptoms or worsening	2 (5.7)

**Table 3:** VAS and ODI improvements.

Follow-up	Median (range)	*P* value
VAS of leg (0-10)		
Preoperation	6.91 (5-8)	
1 day after the operation	1.69 (1-3)	<0.01
1 month after the operation	1.23 (0-2)	<0.01
12 months after the operation	0.91 (0-2)	<0.01
VAS of back (0-10)		
Preoperation	4.51 (3-6)	
1 day after the operation	1.77 (1-3)	<0.01
1 month after the operation	0.86 (0-2)	<0.01
12 months after the operation	0.66 (0-2)	<0.01
ODI (0-100)		
Preoperation	63.82 (52-76)	
1 day after the operation	38.79 (28-52)	<0.01
1 month after the operation	24.79 (18-32)	<0.01
12 months after the operation	11.33 (6-22)	<0.01

**Table 4:** Walking distance.

Outcome (meters)	Preoperation	Postoperation	*P* value
<50	9	0	<0.01
>50 and <500	26	2	<0.01
>500	0	33	<0.01

**Table 5:** Standing time.

Outcome (min)	Preoperation	Postoperation	*P* value
<5	31	3	<0.01
>5 and <15	4	11	<0.01
>15	0	21	<0.01

## Data Availability

The data used to support the findings of this study are available from the corresponding author upon request.
